# Increased incidence of head and neck cancer in liver transplant recipients: a meta-analysis

**DOI:** 10.1186/1471-2407-14-776

**Published:** 2014-10-22

**Authors:** Qian Liu, Lifeng Yan, Cheng Xu, Aihua Gu, Peng Zhao, Zhao-Yan Jiang

**Affiliations:** State Key Laboratory of Reproductive Medicine, Institute of Toxicology, Nanjing Medical University, Nanjing, 211166 China; Key Laboratory of Modern Toxicology of Ministry of Education, School of Public Health, Nanjing Medical University, No. 818 Tianyuan East Road, Nanjing, 211166 China; Department of Neurosurgery, The First Affiliated Hospital, Nanjing Medical University, Nanjing, China; Department of Surgery, Shanghai Institute of Digestive Surgery, Ruijin Hospital, Shanghai JiaoTong University School of Medicine, No. 197 Ruijin Er Road, Shanghai, 200025 China

**Keywords:** Liver transplantation, Head and neck cancer, Standardized incidence ratio, Meta-analysis

## Abstract

**Background:**

It is unclear whether liver transplantation is associated with an increased incidence of post-transplant head and neck cancer. This comprehensive meta-analysis evaluated the association between liver transplantation and the risk of head and neck cancer using data from all available studies.

**Methods:**

PubMed and Web of Science were systematically searched to identify all relevant publications up to March 2014. Standardized incidence ratio (SIR) and 95% confidence intervals (CIs) for risk of head and neck cancer in liver transplant recipients were calculated. Tests for heterogeneity, sensitivity, and publishing bias were also performed.

**Result:**

Of the 964 identified articles, 10 were deemed eligible. These studies included data on 56,507 patients with a total follow-up of 129,448.9 patient-years. SIR for head and neck cancer was 3.836-fold higher (95% CI 2.754–4.918, P = 0.000) in liver transplant recipients than in the general population. No heterogeneity or publication bias was observed. Sensitivity analysis indicated that omission of any of the studies resulted in an SIR for head and neck cancer between 3.488 (95% CI: 2.379–4.598) and 4.306 (95% CI: 3.020–5.592).

**Conclusions:**

Liver transplant recipients are at higher risk of developing head and neck cancer than the general population.

## Background

Liver transplantation is regarded as the therapeutic option of choice in patients with acute and chronic liver disease or liver failure [1]. Long-term outcomes can be enhanced when graft rejection is successfully controlled using immunosuppressive regimens [2, 3]. However, long-term immunosuppression can impair immune function [4], increasing the risk of developing *de novo* malignancies after organ transplantation [5]. The most common of these new malignancies are lymphomas, followed by skin malignancies, cervical carcinoma, renal cancer, vulvar carcinoma and Kaposi’s sarcoma [6–8].

Despite the rarity of head and neck cancers in the general population [6], approximately half of all post-transplant malignancies have been found in sites in the head and neck [4]. The association of between liver transplantation and the risk of developing head and neck cancer has been explored in several populations [9]. Large retrospective trials have found that the incidence of head and neck cancer in liver transplantation recipients ranged from 0.1–2% [10, 11], and that these tumors occurred as early as 34 months after liver transplantation [12–16]. Because of differences in study design, sample selection, sample size, and follow-up period, the association between liver transplantation and head and neck cancer remains unclear. This meta-analysis, which included all relevant studies on head and neck cancer and liver transplantation, was therefore performed to clarify whether the total standardized incidence rate (SIR) of head and neck cancer is higher following liver transplantation than in the general population.

## Methods

### Publication search

A systematic, comprehensive literature search was carried out using the PubMed and Web of Science databases, to identify all articles investigating the risk of head and neck cancer in liver transplant recipients (last search updated on 31 March, 2014). Search keywords, both free text and medical subject headings (MeSH), included ‘liver transplantation’, ‘organ transplantation’, and ‘head and neck cancer’. The search was limited to studies published in English and to those including only human subjects. Abstracts and unpublished reports were not considered.

### Inclusion and exclusion criteria

Studies were selected for meta-analysis if: (a) they were population-based cohort studies in liver transplant recipients; (b) SIR with 95% confidence intervals (CI) were calculated in transplant recipients relative to the general population; (c) they included sufficient data on the incidence, SIR or relative risk (RR) of head and neck cancer, or sufficient numbers of patients; (d) and if they assessed patients with head and neck cancer (ICD: C00-14, C30-32), defined as cancer of the lips, oral cavity, larynx, pharynx, nose and ear. Studies were excluded if: (a) they were case-control studies, case series, or case reports; (b) they lacked sufficient data for meta-analysis; (c) they assessed head and neck cancer following transplantation of organs other than the liver; or (d) they assessed cancers other than head and neck cancer following liver transplantation.

### Data extraction

The data from all qualified and adaptive publications were independently extracted by two investigators (QL and LY) using the inclusion criteria mentioned above; any discrepancies were resolved by discussion. Characteristics extracted from each study included the first author’s family name, year of publication, type of transplant, data source, the country of participants, number of patients, number of liver transplant cases, number of all cancers, length of follow-up time, mean follow-up time (years), patient-years (years), mean age at transplantation (years), median time to development of any type of cancer (months), number of expected cases of head and neck cancer, number of identified cases of head and neck cancer, and the SIRs of commonly known cancers and head and neck cancer.

### Statistical analysis

All statistical analyses were performed using STATA software (version 11; STATA Corporation, College Station, Texas). The unadjusted RR, along with the 95% CI, in each study was employed to assess the strength of head and neck cancer risks in liver transplant recipients relative to the general population.

Heterogeneity among studies, defined as differences in study outcomes, were assessed using a chi-squared-based Q-statistics test. The models of analysis for the pooled RRs were based on the *P* value. A *P* value for the Q-statistic greater than 0.05 indicated a lack of heterogeneity among studies, allowing the use of a fixed-effects model (the Mantel–Haenszel method). Otherwise, a random-effects model (the DerSimonian and Laird method) was applied. *I*^2^ was defined as the proportion of total variation resulting from heterogeneity among studies, as opposed to random error or chance, with *I*^2^< 25%, 25–75% and >75% representing low, moderate and high degrees of inconsistency, respectively [17, 18].

One-way sensitivity analysis was performed to assess the effects of individual study data on the pooled RR. Each included study was removed from the pool and the remaining studies re-analyzed to estimate the stability of the results. Begg’s funnel plot and Egger’s test were used to analyze publication bias. Funnel plot asymmetry was assessed using Egger’s linear regression method on the natural logarithm scale of the RR. A symmetric plot suggested publication bias, with a P value <0.05 indicating significant publication bias.

## Results

### Characteristics of the eligible studies assessed by meta-analysis

**Figure 1:**
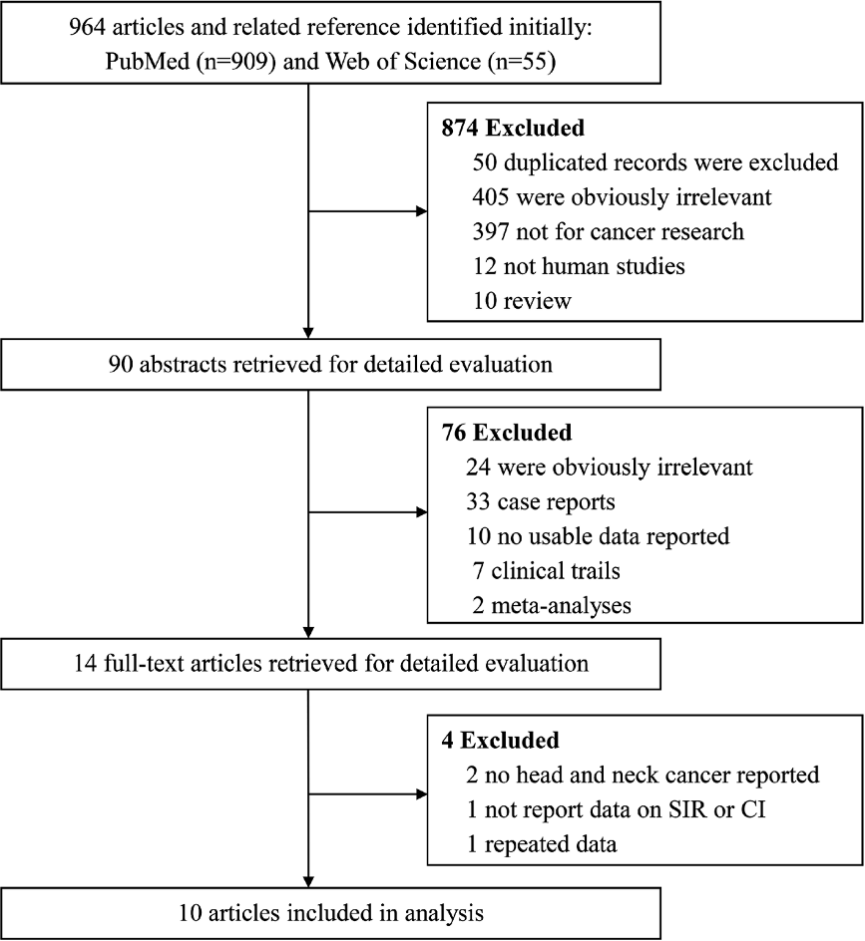
**Literature search and study selection protocol used in this meta-** **analysis.**

The initial search identified 964 related articles. Application of the inclusion and exclusion criteria resulted in the retrieval of 10 eligible publications. These 10 articles, which included 13,853 liver transplants in 56,507 patients, evaluated whether the total SIR for head and neck cancer was higher in liver transplant recipients than in the general population [19–29]. An outline of the literature search and selection process is shown in Figure 1, and the key characteristics of the selected studies are summarized in Table 1. These eligible studies were based on patients in several countries, including Finland, Italy, the UK, the Netherlands, Spain, Canada, Sweden and Japan.

**Table 1:** **Summary of studies included in the analysis**

Study	Year	Type of transplant	Data source	Geographic origin	Number of patients (n)	Number of liver transplant cases
**Ettorre**	2013	Liver	Italian transplantation centers	Italy	1675	1675
**Kaneko**	2013	Liver	Not mentioned	Japan	360	360
**Krynitz**	2012	Multiorgan	Swedish National Patient Register	Sweden	10476	1221
**Baccarani**	2010	Liver	Italian liver transplant centers	Italy	417	417
**Collett**	2010	Multiorgan	UK Transplant Registry	UK	37617	6771
**Herrero**	2010	Liver	Not mentioned	Spain	339	339
**Åberg**	2008	Liver	Finnish liver transplant registry and patient records	Finland	540	540
**Jiang**	2008	Liver	Canadian Organ Replacement Registry	Canada	2034	2034
**Serraino**	2007	Multiorgan	Not mentioned	Italy	2875	322
**Haagsma**	2001	Liver	Medical records	Netherlands	174	174
**Total**	—	—	—	—	**56507**	**13853**

Table 2 shows a summary of the interrelated studies with their demographic details. Of the 545 liver transplant recipients who developed cancers and hematological malignancies, 78 developed head and neck cancer, as compared with 10 expected cases. Mean age at transplantation was 48.83 years (range: 43.00–56.05 years) and median time to develop any type of cancer was 50.1 months (range: 38.4–61.0 months), with a mean follow-up time of 7.3 years (range: 5.1–16.0 years) and a total of 129,448.9 patient-years.

**Table 2:** **Demographic details of patients in the included studies**

Study	Year	Length of follow-up time	Number of all cancers (n)	Mean follow-up time(years)	Patient-years (years)	Mean age at transplantation (years)	Number of expected cases of head and neck cancer	Number of identified cases of head and neck cancer	Median time in months to development of any type of cancer
**Ettorre**	2013	1990–2008	100	5.2	10104.3	53.50	—	19	38.4
**Kaneko**	2013	—	27	7.5		56.00	0.014	1	—
**Krynitz**	2012	1970–2008	150	5.1	93432.0	—	—	6	—
**Baccarani**	2010	1991–2005	43	6.7/6.9	2856.0	52.00	1.330	8	51.0
**Collett**	2010	1980–2007	—	16.0	—	—	—	18	—
**Herrero**	2010	1990–2009	29	7.5	2533.0	—	2.312	8	—
**Åberg**	2008	1982–2005	39	6.3	3222.0	43.00	0.180	2	61.0
**Jiang**	2008	1983–1998	113	—	10370.6	—	1.200	3	—
**Serraino**	2007	—	23	6.5	6931.0	45.50	—	4	—
**Haagsma**	2001	1979–1996	21	5.1	—	43.00	3.310	9	—
**Total**	—	—	545	7.3	129448.9	48.83	8.346	78	50.1

### Evidence synthesis

**Figure 2:**
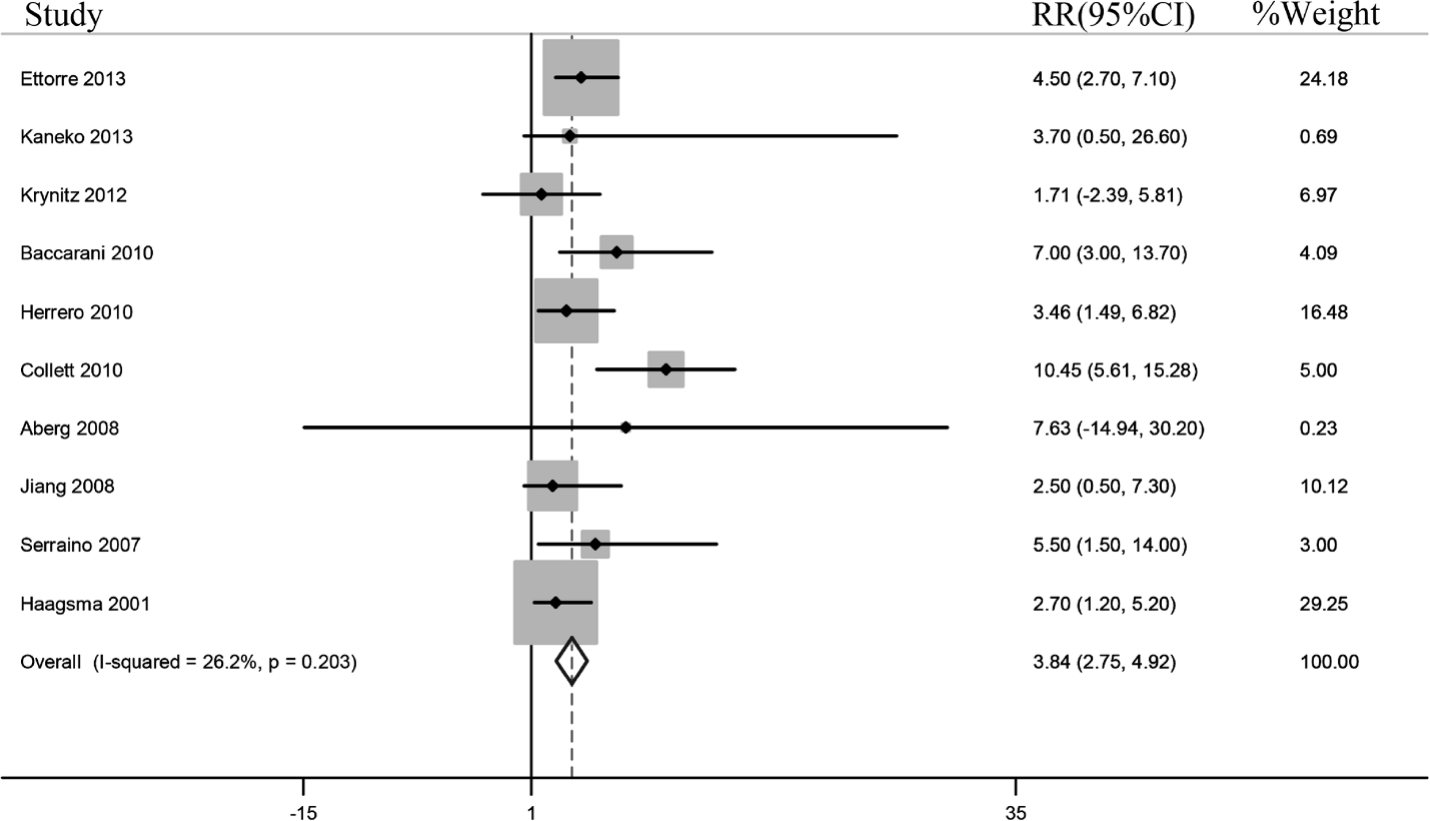
**Forest plots of the relative ratios** **(RRs)** **and 95%** **confidence intervals** **(CIs)** **for overall risk of head and neck cancer.** The squares and horizontal lines correspond to the study-specific RR and 95% CI. The area of the squares reflects the study specific weight.

Calculation of the overall SIR of the 10 articles in our meta-analysis showed that the risk of head and neck cancer was significantly higher in liver transplant recipients than in the general population (SIR = 3.836, 95% CI: 2.754 – 4.918; P = 0.000; Table 3). Three of these studies calculated SIRs for cancers at different sites in the head and neck, including the pharynx, oral cavity and lip [27]; oral cavity and lip [23]; and larynx, pharynx, oral cavity, lip, nose and middle ear [21]. Figure 2 shows forest plots for individual and overall RR measures. There was no heterogeneity (*I*^*2*^ = 26.2%, P_heterogeneity_ =0.203) in the pooled analysis.

**Table 3:** **Overall SIR and 95**% **CI in our meta**-**analysis**

Study	Year	SIR	95% Confidence intervals	
			LCI	UCI
**Ettorre**	2013	4.500	2.700	7.100
**Kaneko**	2013	3.700	0.500	26.600
Krynitz*	2012	1.710	-2.387	5.808
**Baccarani**	2010	7.000	3.000	13.700
**Herrero**	2010	3.460	1.490	6.820
Collett*	2010	10.446	5.608	15.285
Aberg*	2008	7.628	-14.943	30.199
**Jiang**	2008	2.500	0.500	7.300
**Serraino**	2007	5.500	1.500	14.000
**Haagsma**	2001	2.700	1.200	5.200
**Combined**	—	**3.836**	**2.754**	**4.918**

*Combined data of more than one cancer site.

Table 4 shows that the risks of cancers other than head and neck cancer, including non-Hodgkin lymphoma (NHL), Hodgkin lymphoma, non-melanoma, kidney, lung, esophagus and Kaposi’s sarcoma were also significantly increased in liver transplant recipients.

**Table 4:** **Cancer**-**specific standardized incidence ratios** (**SIRs**)

Cancer site	Aberg 2008	Baccarani 2010	Collett 2010	Ettorre 2013	Haagsma 2001	Jiang 2008	Krynitz 2012	Serraino 2007	Kaneko 2013	Herrero 2010	Overall SIR
**Head and neck**	7.63(-14.94-30.20)	7.0 (3.0-13.7)	10.45(5.61-15.29)	4.5 (2.7-7.1)	2.7 (1.2-5.2)	2.5 (0.5-7.3)	1.71(-2.39-5.81)	5.5 (1.5-14)	3.7 (0.5-26.6)	3.46 (1.49-6.82)	**3.836** **(2.754**–**4.918)**
**NHL**	13.9 (6.01-27.4)	13.8 (6.3–26.2)	13.3(10.6,16.6)	7.1 (4.2-11.2)	—	20.8(14.9-28.3)	14 (8.9–21)	16.4 (5.3–38.2)	—	—	**12.174** **(10.231–** **14.117)**
**Hodgkin lymphoma**	14.7 (0.37-82.0)	—	8.9(3.8,17.5)	6.3 (0.8-22.9)	—	—	0.0 (0.0–27)	—	—	—	**7.027** **(1.727**–**12.328)**
**Leukemia**	0.00 (0.00-41.4)	—	—	—	—	3.9 (1.0-9.9)	4.5 (0.5–16)	—	15.1(4.9-46.9)	—	4.266 (0.533–8.000)
**Melanoma of the skin**	2.10 (0.05-11.7)	4.4 (0.5–16.1)	—	3.1 (0.9-8.1)	—	—	1.5 (0.3–4.5)	—	—	—	2.040 (0.349–3.730)
**Nonmelanoma**	38.5 (18.5-70.8)	—	6.6(5.8,7.5)	—	70.0(28.1-144.2)	—	—	—	6.4 (1.6-25.4)	—	**6.646** **(5.799**–**7.493)**
**Liver**	0.00 (0.00-28.4)	—	—	0.8 (0.2-2.3)	—	—	14 (7.0–27)	0.0 (0.0–10.9)	—	—	0.906 (-0.117–1.929)
**Pancreas**	2.31 (0.06-12.9)	—	—	1.1 (0.1-3.9)	—	3.3 (0.7-9.6)	3.6 (0.7–11)	—	—	—	1.703 (0.100–3.305)
**Kidney**	4.17 (0.50-15.1)	—	1.8(0.8,3.6)	—	30.0 (6.1-87.7)	3.1 (0.8-7.9)	1.9 (0.2–6.9)	—	6.4 (1.6-25.4)	3.31(1.51-6.28)	**2.335** **(1.270**–**3.401)**
**Lung,** **trachea**	0.00 (0.00-3.32)	1.6 (0.4–4.1)	1.6(1.2,2.2)	1.1 (0.6-1.9)	—	1.4 (0.7-2.6)	1.8 (0.7–4.0)	0.5 (0.0–3.1)	—	2.17(0.99-4.12)	**1.371** **(1.043**–**1.698)**
**Breast**	0.26 (0.01-1.43)	0.6 (0.0–3.4)	0.8(0.5,1.1)	0.7 (0.1-1.9)	—	0.6 (0.2-1.4)	1.0 (0.4–2.1)	—	0.9 (0.1-6.4)	—	0.719 (0.489–0.949)
**Prostate**	1.24 (0.15-4.47)	—	—	—	—	1.0 (0.3-2.4)	0.5 (0.1–1.2)	—	2.2 (0.6-8.9)	—	0.658 (0.186–1.131)
**Stomach**	4.97 (0.60-18.0)	3.7 (0.8–10.9)	—	0.6 (0.1-2.1)	—	—	3.9 (0.8–11)	—	1.6 (0.6-4.3)	—	1.033 (0.182–1.883)
**Esophagus**	—	23.4 (7.6–54.7)	—	—	—	—	2.6 (0.1–15)	—	16.9(2.4-17.9)	7.91(1.63-23.13)	**9.726** **(5.018**–**14.433)**
**Kaposi’** **s sarcoma**	—	144.0(52.9–313.5)	—	37.3(13.7-81.2)	—	—	80 (2.0–444)	—	—	—	**44.778** **(12.458**–**77.099)**
**Cervix uteri**	—	30.7 (6.3–89.8)	—	—	—	—	2.6 (0.1–15)	—	—	—	3.467 (-3.867–10.801)
**Thyroid gland**	0.00 (0.00-12.7)	—	20.0(5.4,51.2)	—	—	—	0.0 (0.0–14)	—	—	—	0.809 (-3.798–5.416)
**All cancer**	0.46 (-1.5–1.07)	1.81 (0.65–2.975)	**1.6** **(1.36**–**1.84)**	1.13(0.72–1.53)	2.85 (0.85–4.84)	1.07(0.62–1.51)	0.94 (0.52–1.36)	0.86(-0.59–2.30)	2.27(0.84–3.70)	**2.76** **(1.59**–**3.93)**	**1.319** **(1.159**–**1.478)**

Bold data represent positive results.

### Sensitivity analysis

**Figure 3:**
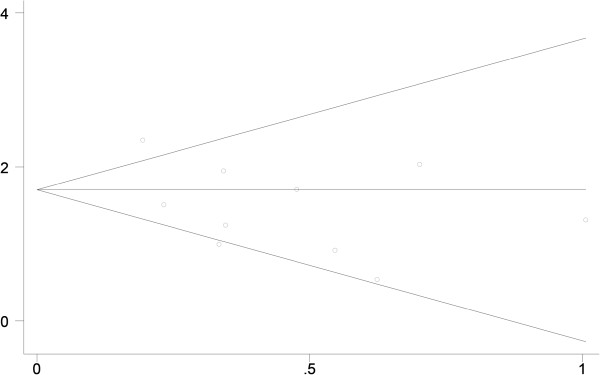
**Results of sensitivity analysis examining head and neck cancer risk in liver transplant recipients.**

The sensitivity analysis assessed the influence of an individual study on the pooled RR by omitting one study and re-analyzing the results. In assessing the association of liver transplantation with the risk of head and neck cancer risk, we found that no individual study altered the overall significance of the RRs, suggesting the stability and reliability of the overall results (Figure 3). Sensitivity analysis indicated that the omission of any one study resulted in SIRs between 3.488 (95% CI: 2.379–4.598) and 4.306 (95% CI: 3.020–5.592; Table 5).

**Table 5:** **Sensitivity analysis**

Study	Year	RR	95% Confidence intervals	
			LCI	UCI
**Ettorre**	2013	3.625	2.382	4.867
**Kaneko**	2013	3.837	2.752	4.923
**Krynitz**	2012	4.000	2.874	5.117
**Baccarani**	2010	3.701	2.597	4.806
**Herrero**	2010	3.910	2.727	5.094
**Collett**	2010	3.488	2.379	4.598
**Aberg**	2008	3.827	2.744	4.910
**Jiang**	2008	3.986	2.846	5.128
**Serraino**	2007	3.785	2.687	4.883
**Haagsma**	2001	4.306	3.020	5.592
**Combined**	—	**3.836**	**2.754**	**4.918**

### Publication bias

**Figure 4:**
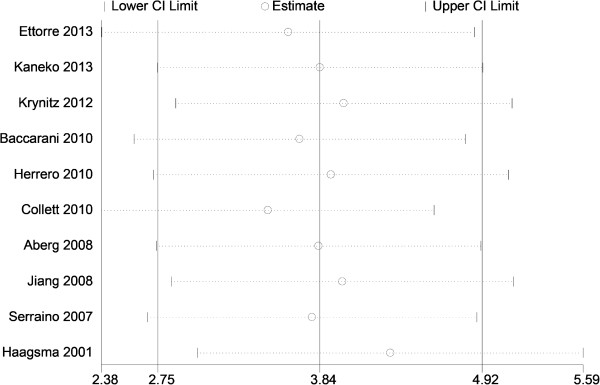
**Begg’** **s funnel plots of head and neck cancer risk across all populations.**

Potential publication bias of the literature was assessed using Funnel plots and Begg’s and Egger’s tests. Funnel plot asymmetry was not observed. Publication bias was not evident (t = -1.72, *P* = 0.124) (Figure 4).

## Discussion

Liver transplant recipients may have increased risk for developing *de novo* cancers [30], with most of these cancers appearing about 6 years after transplantation [3]. Tumors such as NHL and Kaposi’s sarcoma, which are rare in the general population, are encountered more frequently in transplant recipients. Head and neck cancer, which accounts for <4% of malignancies in the general population, accounts for about 15% of malignancies in transplant recipients [4, 31]. This meta-analysis was performed to clarify the relationship between head and neck cancer and liver transplantation, finding that transplant recipients were at significantly greater risk of head and neck cancer than the general population (SIR = 3.836, 95% CI 2.754–4.918, P = 0.000). Thus, these results provide further evidence that organ transplant recipients may be at increased risk of head and neck cancer compared with the general population.

The exact mechanism associated with the increased incidence of *de novo* head and neck cancer in liver transplantation recipients is unknown. Long-term administration of immunosuppressive agents may be responsible, as reported for other malignancies [4, 32, 33]. These immunosuppressive agents may depress immune surveillance and permit tumor development [29, 34]. Indeed, immunosuppression resulting from genetic defects, AIDS or drugs, has been associated with a higher risk of malignancy than in an immunocompetent population [35]. Anti-rejection reagents, such as azathioprine, CyA and tacrolimus, may favor the establishment of a *de novo* malignancy by initiating and/or potentiating DNA damage [36]. Alternatively, these agents may induce phenotypic changes in cells including nontransformed cells, increasing membrane ruffling, cell locomotion, and extracellular matrix-independent growth mediated by transforming-growth-factor [37]. Tacrolimus, which is a more potent immunosuppressive agent than CyA, showed a dose-dependent relationship with *de novo* malignancy [38]. Alcohol intake, which has been associated with tumor development in patients with alcoholic liver cirrhosis and after liver transplantation, may also be responsible [22]. Alcoholic liver cirrhosis is a common chronic liver disease and an indication for liver transplantation [39]. The incidence of *de novo* malignancies was found to be significantly higher in patients who underwent liver transplantation for alcoholic than for non-alcoholic cirrhosis [40]. Alcohol consumption is also an established risk factor for head and neck cancers [41]. Interestingly, oropharyngeal carcinoma was observed only in patients who underwent liver transplantation for alcoholic liver cirrhosis [42]. However, the articles included in our meta-analysis did not include data on alcohol consumption in liver transplant recipients, preventing a determination of whether liver transplantation was independently associated with a higher risk of head and neck cancer. Previous studies have also demonstrated the significance of persisting pre-transplantation tobacco abuse on post-transplantation outcomes [43]. In contrast, quitting smoking after liver transplantation might protect against tumor growth owing to the association between tobacco abuse on neoplasia [22]. Disease-free survival was worse in liver transplant patients with head and neck cancer and a history of smoking (active or prior smokers) than in nonsmokers [44]. The association between tobacco abuse and poor health outcomes suggests the need for liver transplant candidates to quit smoking. Interventional and screening programs can help reduce transplant-related tumorigenesis in liver transplant recipients. Several other risk factors are associated with malignancy in liver transplant recipients, including adverse psychosocial factors, poor treatment modality, and premalignant disease [30, 44, 45]. A systematic review has com-prehensively analyzed these risk factors [30].

This study had several limitations: 1) Most of the studies were from western countries, with only one involving patients from an Asian country (Japan). Despite an exhaustive literature search and the absence of publication bias, as shown by the funnel plot and Egger’s test, some non-English publications and unpublished data related to our study may have been omitted, especially those reporting negative findings that could potentially influence the results. It was not possible to evaluate the effect of ethnicity (genetic background) on the association between head and neck cancer and liver transplantation. 2) The liver transplant recipients were not all screened for head and neck cancer before transplantation. Therefore, we could not exclude the possibility that some of these cancers may have been present prior to transplantation. 3) Because our screened studies had patients of different backgrounds, and their methods and sample size varied, some data were missing. Two indices, mean age at diagnosis of malignancy and mean time to develop head and neck cancer, were not measured in the included studies. 4) Detailed information on the type and dosage of immunosuppressive drugs was not available for further subgroup analysis. Low-dose cyclosporine was associated with a lower rate of malignancy than high-dose cyclosporine in renal transplant recipients [46]. Moreover, tacrolimus is more potent than cyclosporine. Different agents may exert different effect on the development of cancers. 5) Transplant recipients may be followed up more regularly here than the general population. However, these findings are not available. Despite these limitations, our study did not show significant heterogeneity or publication bias, indicating that these results were reliable.

## Conclusions

Our meta-analysis showed that liver transplant recipients were at a higher risk of developing head and neck cancer than the general population. Liver transplant recipients should therefore be screened intensively for head and neck cancer, which may enhance early diagnosis and treatment, as well as patient outcomes.
